# NIRS as a tool for assaying emotional function in the prefrontal cortex

**DOI:** 10.3389/fnhum.2013.00770

**Published:** 2013-11-18

**Authors:** Hirokazu Doi, Shota Nishitani, Kazuyuki Shinohara

**Affiliations:** Graduate School of Biomedical Sciences, Nagasaki UniversityNagasaki, Japan

**Keywords:** NIRS, emotion, prefrontal cortex, hemispheric asymmetry, reward, autonomic nervous system

## Abstract

Despite having relatively poor spatial and temporal resolution, near-infrared spectroscopy (NIRS) has several methodological advantages compared with other non-invasive measurements of neural activation. For instance, the unique characteristics of NIRS give it potential as a tool for investigating the role of the prefrontal cortex (PFC) in emotion processing. However, there are several obstacles in the application of NIRS to emotion research. In this mini-review, we discuss the findings of studies that used NIRS to assess the effects of PFC activation on emotion. Specifically, we address the methodological challenges of NIRS measurement with respect to the field of emotion research, and consider potential strategies for mitigating these problems. In addition, we show that two fields of research, investigating (i) biological predisposition influencing PFC responses to emotional stimuli and (ii) neural mechanisms underlying the bi-directional interaction between emotion and action, have much to gain from the use of NIRS. With the present article, we aim to lay the foundation for the application of NIRS to the above-mentioned fields of emotion research.

## INTRODUCTION

Since being introduced as a research tool, near-infrared spectroscopy (NIRS) has gained wide support and recognition among cognitive neuroscientists, despite having several disadvantages when compared with other non-invasive measurements of neural activation. For instance, NIRS has poor spatial resolution compared with other neuroimaging techniques that measure neurovascular response, such as functional magnetic resonance imaging and positron emission tomography. Similarly, the temporal resolution of NIRS is much lower than that of electroencephalography (EEG) and magnetoencephalography.

The acceptance of NIRS as a novel technique for measuring neural activation might be partly attributable to several unique characteristics. First, NIRS measurement is thought to impose a considerably less severe physical and psychological burden than that of existing neuroimaging techniques. Thus, this technique is particularly advantageous for measuring neural responses in the elderly and infantile populations (Ichikawa et al., 2010; Ozawa et al., 2011; Kida and Shinohara, 2013b). Second, the ease of NIRS measurement makes it a suitable technique for collecting data from a large participant cohort. Third, the measurement of neural activation using near-infrared light is, in principle, more robust with respect to exogenous noise in the environment. Thus, NIRS is considered to be a useful technique for measuring neural activation under less constrained and more ecologically valid settings (Tuscan et al., 2013).

During the past two decades, a number of researchers have used NIRS to produce novel insights about the neural mechanisms underlying various cognitive and perceptual functions. At the same time, the above-mentioned methodological advantages of NIRS have not been fully exploited. For example, the majority of existing NIRS studies measured brain activation under severely structured settings, with several exceptions (Suzuki et al., 2004).

One field of research, that has much to gain from the use of NIRS, is emotion research. However, there are several concerns that impact the efficacious use of NIRS in this field. In the first part of this mini-review, we discuss findings from NIRS studies on emotion processing with respect to existing views on emotional function in the prefrontal cortex (PFC). Due to the widely accepted convention of functional NIRS studies, we tentatively treat the increase in the oxygenated-hemoglobin concentration [referred to as (oxy-Hb)] as the primary and genuine indicator of cortical activation in this review. At the same time, the possibility that the [oxy-Hb] change reflects the peripheral responses other than the neural activation is discussed in the later part. In the second half of the article, we describe a potential avenue of emotion research where the unique characteristics of NIRS could be gainfully used. We also discuss several practical problems that researchers might face when conducting emotion research using NIRS. This article is not intended to serve as a comprehensive archive of previous findings. Rather, the goal of this article is to lay a foundation for the use of NIRS in several areas of emotion research.

### EMOTION PROCESSING IN THE PFC

The neural mechanisms underlying emotional experience and mood have been the focus of intensive research in both the fields of cognitive neuroscience and clinical psychiatry. According to the now classic “limbic system” model (MacLean, 1949) of neural mechanisms of emotional response, evolutionally ancient subcortical structures generate primitive emotions, such as fear. Emotions originating in the “reptilian brain” are further elaborated in the diverse brain regions of phylogenetically advanced neural circuits, including the PFC. Consistent with this model, more recent studies have identified the PFC as a key region in the induction and regulation of emotional responses (Davidson and Fox, 1982; Damasio, 1996; Rolls, 1996).****

Although there is now a wealth of empirical evidence for the role of the PFC in emotion processing, the exact function subserved by this region is unclear. At the same time, there are several widely accepted views regarding the function of each subregion of the PFC, as summarized in an insightful review by Dalgleish (2004). First, the orbitofrontal region of the PFC has been closely linked to reward processing and reinforcement learning (Rolls, 1996). Specifically, the orbitofrontal PFC appears to play a pivotal role in associating exogenous stimuli with rewarding reinforcers, thereby promoting the assignment of emotional value and saliency. Second, the ventromedial PFC may act as an interface between visceral reactions and higher cognitive function. This view is championed by the influential “somatic-marker hypothesis” (Damasio, 1996), which proposes that somatic markers, as peripheral reactions to stimuli, are processed in the ventromedial PFC as part of a system that guides higher order cognitive functions. Third, the “valence asymmetry hypothesis” of the PFC (Davidson et al., 1990) suggests that the motivational tendency of a living organism can be conceptualized along the dimension of approach/withdrawal. More specifically, when approach motivation is activated, an organism is strongly motivated to pursue an appetitive or rewarding goal. Contrarily, the activation of withdrawal motivation emphasizes avoidance of harmful situations rather than acquisition of rewards. The core proposition of the valence asymmetry hypothesis is that the right PFC activates withdrawal motivation and the left PFC activates approach motivation, thereby enabling adaptive behaviors.

## OVERVIEW OF EXISTING NIRS STUDIES

Several NIRS studies have examined the role of PFC activation in emotion processing. An overview of these findings could be beneficial for several reasons. First, the existing findings might serve as scaffolding upon which researchers could build novel experimental designs. Second, a summary of existing NIRS data might support or oppose established views about the emotional function of the PFC (Davidson and Fox, 1982; Damasio, 1996; Rolls, 1996). Although different types of hemodynamic response are frequently treated as equal [oxy-Hb] reflects different aspects of task-related hemodynamic responses from the concentration of deoxygenated hemoglobin [referred to as (deoxy-Hb)] whose change is supposed to be closely linked to BOLD response (Song et al., 2006). Therefore, a close examination of NIRS data might produce a more comprehensive picture about neural activation during emotion processing. In the following sections, we briefly review the previous findings in light of the above-mentioned theories about the nature of emotion processing in the PFC (Davidson and Fox, 1982; Damasio, 1996; Rolls, 1996). The details about the major studies covered below are summarized in **Table 1**.

**Table 1:** Summary of the findings of the major NIRS studies covered in the present review.

Reference	Participants	Task	Major findings
Kida and Shinohara (2013b)	Adults	Tactile stimulation by velvet	Increased [oxy-Hb] to velvet in the bilateral anterior PFC
Minagawa-Kawai et al. (2009a)	Mothers and her infants	Passive viewing of smiling faces	Increased [oxy-Hb] in the OFC region in response to own mother/infant’s smiling face in both mothers and infants
Kida and Shinohara (2013a)	3, 6, 10 month-olds	Tactile stimulation by wood-packed velvet to the left palm	Bilateral increase of [oxy-Hb] in the anterior PFC by velvet stimulation only in 10 month-olds
Tanida et al. (2007)	Young adult females	Stress induction by mental arithmetic	Right lateralized increase in [oxy-Hb] being linked to ANS activation and skin conditions
Matsuo et al. (2003)	Victims of traumatic event with or without PTSD	Passive viewing of trauma related video clips	Large and long-lasting increase of [oxy-Hb] concomitant with decrease of [deoxy-Hb] in the DLPFC in victims with PTSD
Moghimi et al. (2012)	Adults	Presentation of emotional music excerpts	Music excerpts rated as intense induced larger peaks of [oxy-Hb] change. The sharpness of [oxy-Hb] peak was also linked to arousal and valence ratings
Morinaga et al. (2007)	Adults	Anticipation of electrical shock	Increased [oxy-Hb] during the anticipation of electrical shock in the right PFC
Leon-Carrion et al. (2006)	Adults	Presentation of emotional video clips	Pronounced gender difference in [oxy-Hb] change after the offset of emotional video clips

### SENSITIVITY TO REWARDING STIMULI

As for the reward sensitivity of the PFC (Rolls, 1996), at least two studies with adult participants have found that [oxy-Hb] in the vicinity of the orbitofrontal region of the PFC increases following exposure to rewarding stimuli, such as tactile stimulation by velvet (Kida and Shinohara, 2013a) and viewing one’s infant smiling (Minagawa-Kawai et al., 2009a). Interestingly, an analogous increase in [oxy-Hb] has also been observed in the same region in infants (Minagawa-Kawai et al., 2009a; Kida and Shinohara, 2013b), suggesting that NIRS is a suitable method for measuring reward system activation in participants of varying ages.

### PROCESSING OF VISCERAL REACTIONS

Few NIRS studies to date have specifically examined the neural mechanisms mediating the influence of visceral “somatic” markers on behavior. This is partly due to a technical limitation of NIRS. The ventromedial PFC, which is considered to be the locus of integration between somatic markers and higher order cognitive functions (Damasio, 1996), is located too far from the cranium surface for accurate measurements of activation using NIRS.

With regard to the link between visceral reactions and the PFC, several NIRS studies have succeeded in revealing an association between activation of the PFC and activation of the autonomic nervous system (ANS) in response to emotional stimulation. For example, Tanida et al. (2007) reported that the degree of right-lateralized asymmetry in PFC activation patterns observed during mental stress was positively correlated with the level of activation of the sympathetic nervous system. Likewise, increased [oxy-Hb] has been positively correlated with heart rate change when viewing trauma-related video clips (Matsuo et al., 2003). Furthermore, Moghimi et al. (2012) have linked the steepness of the peak of [oxy-Hb] to a subjectively reported arousal level, which is a relatively coarse, but widely accepted indicator of ANS activation (for similar findings, see Matsuo et al., 2003; Roos et al., 2011). These studies offer partial support for the view that the PFC processes visceral reactions, or somatic markers, associated with exogenous stimuli. At the same time, these findings are mere correlational, and so caution should be exercised in interpreting such data. Furthermore, if causal relations are present, the direction of causality has yet to be clarified.

### HEMISPHERIC ASYMMETRY

Many NIRS studies have used bilateral probes to measure hemodynamic responses, and thus have datasets that are suitable for examining hemispheric asymmetry in the PFC. In these studies, either one of the following criteria was adapted to judge the hemispheric asymmetry in the cortical activations; (1) the significant increase of [oxy-Hb] from the baseline in only one of the hemispheres, or (2) the significant inter-hemispheric difference in the level of [oxy-Hb] change. Several studies have produced evidence in support of the valence-asymmetry hypothesis (Morinaga et al., 2007; Marumo et al., 2009; Tuscan et al., 2013). For example, Morinaga et al. (2007) reported that anticipation of an electrical shock was associated with a greater increase in [oxy-Hb] in the right compared with the left PFC. Furthermore, increases in [oxy-Hb] in the right PFC were positively correlated with the strength of harm-avoidance tendencies in the participants. At the same time, a number of studies have failed to detect hemispheric asymmetry in task-related activation as predicted by the valence-asymmetry hypothesis (Herrmann et al., 2003; Kobayashi et al., 2007; Yang et al., 2007; Hoshi et al., 2011). Much of the empirical support for the valence-asymmetry hypothesis has been obtained by measuring asymmetry in EEG alpha power (Davidson and Fox, 1982; Hagemann, 2004). However, the relationship between the EEG power and transient neurovascular response (as measured by NIRS) is not straightforward. Thus, it is possible that phasic changes in [oxy-Hb] are less sensitive than EEG with respect to changes in approach/withdrawal motivation.

## METHODOLOGICAL PROBLEMS IN THE APPLICATION OF NIRS TO EMOTION RESEARCH

A standardized method of analyzing NIRS signals has yet to be established. Aside from the general challenges that researchers face when analyzing NIRS data, the application of NIRS to emotion research requires additional considerations.

The first problem concerns noise caused by peripheral responses to emotional stimulation. The induction of an emotional state is often accompanied by changes in bodily state, such as the contraction of facial muscles or increased cardiovascular activity. Although mitigated by homeostatic regulation, such changes in heart rate and blood pressure could potentially mask task-related hemodynamic responses in NIRS signals. Likewise, the aerobic process of energy consumption associated with muscle contraction may induce significant changes in measurable [oxy-Hb]. Schecklmann et al. (2010) found no systematic relationship between electromyograph signals and [oxy-Hb] during a verbal fluency task. However, the influence of peripheral responses on NIRS signals has been examined only under limited conditions. A regression analysis conducted using simultaneous measurements of NIRS signals and indicators of peripheral response, such as electromyography, heart rate, and blood pressure, could be used to exclude the influence of these factors (Schecklmann et al., 2010).

Another problem is the temporal course of neural activation induced by emotional stimulation. Both the subjective experience of an emotion and the neural responses elicited by emotional stimulation may last longer than the stimulation itself (León-Carrión et al., 2007). Thus, the application of conventional pre-processing methods, such as the correction of global drift (Minagawa-Kawai et al., 2009b) by linear fitting (for example, Takizawa et al., 2008), carries the risk of eliminating important results.

This point was emphasized by Leon-Carrion et al. (2006), who reported that gender differences in cortical activation were present “after” the offset of emotional stimulation. If the researchers had corrected for global drift of the NIRS signal using the period after stimulation offset as the post-stimulation baseline, the observed gender differences may have been obscured. One potential way to address this issue is the use of subjective ratings of emotional state or ANS activation monitoring to continuously track the temporal course of emotional responses for a prolonged duration. This would enable researchers to empirically define the temporal window, and then quantify the hemodynamic responses on the basis of these data.

**FIGURE 1:**
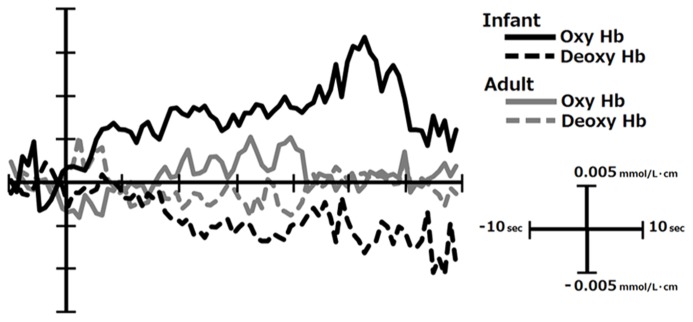
**The temporal course of [oxy-Hb] and [deoxy-Hb] change in response to infants’ and adults’ faces in the right inferior PFC in mothers.** This figure was created from the data reported in Nishitani et al. (2011). The infants’ faces are generally perceived to be more emotionally pleasant and arousing by mothers than those of adults.

The third problem is the selection of the appropriate indicator of the cortical activation. In many of the previous studies, the lasting increase of [oxy-Hb] was taken as the indicator of cortical activation, partly because this parameter is quite sensitive to emotional stimulation as seen from the representative data in **Figure 1** collected in our lab (Nishitani et al., 2011). At the same time, Suh et al. (2006) have reported that the direct cortical stimulation induced vary rapid (within 1–2 s after stimulation) and spatially localized increase in [deoxy-Hb], while the total-hemoglobin concentration change in the later latency-range was only poorly localized. Given this, the conventional analysis method, i.e., averaging the [oxy-Hb] level during the whole stimulation period lasting for several seconds, may have impaired to some extent the power to localize the centroid of cortical activation in the previous research. This may partly explain the failure to find consistent pattern of hemispheric asymmetry in the existing NIRS studies (see Hemispheric Asymmetry). In order to avoid this problem, emotion researchers are advised to statistically evaluate the changes in [deoxy-Hb] as well as [oxy-Hb] with high temporal resolution using analytic methods such as point-by-point testing that is widely conducted in the event-related potential studies (Blair and Karniski, 1993).

## POTENTIAL APPLICATIONS OF NIRS

The above-mentioned theories about the role of the PFC in emotion processing (Davidson and Fox, 1982; Damasio, 1996; Rolls, 1996) all share the view that PFC is one of the key regions where the emotional and motivational reaction bias a wide array of behaviors ranging from attentional allocation, motor function, and decision-making. The importance of PFC function in emotion processing makes it an essential target for future emotion research. In the next section, we present a brief overview of potential fields of research in which NIRS could aid investigations of emotion processing.

### THE BIOLOGICAL BASIS OF INDIVIDUAL DIFFERENCES IN EMOTIONAL RESPONSE

Several recent studies have indicated that traits associated with sensitivity to reward and stress may modulate vulnerability to pathological conditions such as depression (Pizzagalli et al., 2005; Bogdan et al., 2013). These findings give weight to investigations about biological predisposition determining the PFC response to emotion-inducing stimuli. Such research may help elucidate the causes of individual differences in emotional reactions, and potentially help in identifying risk factors that lead to psychiatric conditions. The use of NIRS to measure PFC activation is invaluable due to the suitability of this technique for measuring cortical activation in a large cohort of participants.

For example, the application of NIRS in the field of genetic neuroimaging may enable researchers to acquire data from larger numbers of participants in an economically feasible way, and consequently increase the reliability of their findings. In recent years, there has been a surge in the number of multidisciplinary studies focused on the link between genetic polymorphism and neural function (Hariri et al., 2005; Northoff, 2013). This type of research requires data from a large cohort of participants to mitigate the influence of the confounding variables, such as other genetic predispositions and environmental factors. However, previous fMRI and EEG studies have recruited a modest number of participants (for instance, between 40 and 60), which somewhat compromises the reliability of the existing findings.

### NEURAL SUBSTRATES MEDIATING THE LINK BETWEEN ACTION AND EMOTION

Another important topic related to emotion processing in the PFC is the influence of the emotional state on motor function. As stated by Frijda (1987), one of the primary functions of emotion is to guide adaptive motor behavior. However, until very recently, the notion of “action” has been largely absent in research about the neural underpinnings of emotion, with few exceptions (van Peer et al., 2007; Volman et al., 2011). Motor activity has long been known to directly modify emotion and mood (Niedenthal, 2007), and a bi-directional relationship between motor function and the subjective experience of emotion has been established (Zhu and Thagard, 2002). Considering that the PFC is functionally connected with several motor regions like cerebellum (Kipping et al., 2013) and basal ganglia (Kung et al., 2013), it is likely that this region mediates the complex interactions between motor function and emotion.

Many neuroimaging techniques restrict the diversity of actions that can be performed simultaneously while measurements of neural activation are being collected. This, in turn, limits the types of phenomena that researchers can investigate. As NIRS is robust with respect to external noise, it has potential for widening the scope of investigations of the neural mechanisms underlying the interaction between emotion and action.

## CONCLUSION

Despite technical limitations, NIRS is a reliable technique for quantifying several aspects of emotional functioning in the PFC, such as sensitivity to rewarding stimuli (Rolls, 1996) and processing of visceral reactions (Damasio, 1996). There are some practical challenges when using NIRS to research emotion. However, when adequate measures are taken to address these issues, NIRS is an invaluable tool that has the potential to expand the scope of investigations about the emotional function of the PFC.

## Conflict of Interest Statement

The authors declare that the research was conducted in the absence of any commercial or financial relationships that could be construed as a potential conflict of interest.
